# Draft Genome Sequences of Six *Pseudoalteromonas* Strains, P1-7a, P1-9, P1-13-1a, P1-16-1b, P1-25, and P1-26, Which Induce Larval Settlement and Metamorphosis in *Hydractinia echinata*

**DOI:** 10.1128/genomeA.01477-15

**Published:** 2015-12-17

**Authors:** Jonathan L. Klassen, Thomas Wolf, Maja Rischer, Huijuan Guo, Ekaterina Shelest, Jon Clardy, Christine Beemelmanns

**Affiliations:** a University of Connecticut, Department of Molecular & Cell Biology, Storrs, Connecticut, USA; b Leibniz Institute for Natural Product Research and Infection Biology eV, Jena, Germany; c Harvard Medical School, Department of Biological Chemistry and Molecular Pharmacology, Boston, Massachusetts, USA

## Abstract

To gain a broader understanding of the importance of a surface-associated lifestyle and morphogenic capability, we have assembled and annotated the genome sequences of *Pseudoalteromonas* strains P1-7a, P1-9, P1-13-1a, P1-16-1b, P1-25, and P1-26, isolated from *Hydractinia echinata*. These genomes will allow detailed studies on bacterial factors mediating interkingdom communication.

## GENOME ANNOUNCEMENT

*Pseudoalteromonas* strains P1-7a, P1-9, P1-13-1a, P1-16-1b, P1-25, and P1-26 were isolated from the tissue of a feeding polyp of the marine hydroid *Hydractinia echinata* (1) purchased from the Marine Biological Laboratory in Woods Hole, MA, USA. Pseudoalteromonads are commonly isolated from biofilms of marine surfaces and host tissue of marine invertebrates (2, 3). Their effects on the settlement and metamorphosis of biofouling invertebrates (4–6) and the production of pharmacologically active compounds (7) have been extensively studied. Six *Pseudoalteromonas* strains were isolated from *H. echinata* and screened for their effects on its larval settlement and metamorphosis using a colony-based assay (1). Genomes from the most inductive strains P1-7a, P1-9, P1-13-1a, P1-16-1b, P1-25, and P1‑26 were sequenced to identify candidate genes responsible for larval settlement. Genomic DNA was extracted using the GenElute Blood Genomic DNA kit (Sigma-Aldrich) according to the manufacturer’s protocol. Sequencing performed at the Harvard Medical School Biopolymers Facility used Illumina TruSeq 50 bp single-read libraries and a HiSeq2000 instrument (Illumina CASAVA 1.8.2). After subsampling reads to achieve ~50× coverage, genomes were assembled using the A5 pipeline v20120518 (8) and screened for contamination using blobology (9). Genomes were annotated using Prokka v1.10 (10) and assembly statistics were calculated using scripts from the Assemblathon2 project (11).

The draft genome sequence of strain P1-7a was sequenced to 52× coverage, and comprises 189 contigs totaling 4,374,565 bases in length and having a G+C content of 40.8%. Its annotation includes 3,853 coding sequences (CDSs), 96 tRNAs, and 4 rRNAs.

The draft genome of strain P1-9 was sequenced to 47× coverage, and comprises 211 contigs totaling 4,808,111 bases in length and having a G+C content of 40.7%. Its annotation includes 4,321 CDSs, 84 tRNAs, and 3 rRNAs.

The draft genome sequence of strain P1-13-1a was sequenced to 51× coverage, and comprises 174 contigs totaling 4,442,776 bases in length and having a G+C content of 40.7%. Its annotation includes 3,930 CDSs, 93 tRNAs, and 3 rRNAs.

The draft genome of strain P1-16-1b was sequenced to 57× coverage, and comprises 90 contigs totaling 3,977,637 bases in length and having a G+C content of 40.1%. Its annotation includes 3,562 CDSs, 90 tRNAs, and 4 rRNAs.

The draft genome sequence of strain P1-25 was sequenced to 51× coverage, and comprises 163 contigs totaling 4,399,610 bases in length and having a G+C content of 40.7%. Its annotation includes 3,855 CDS, 97 tRNAs, and 3 rRNAs.

The draft genome sequence of strain P1-26 was sequenced to 48× coverage, and comprises 219 contigs totaling 4,715,935 bases in length and having a G+C content of 41.2%. Its annotation includes 4,183 CDS, 96 tRNAs, and 4 rRNAs.

Genes associated with secretion (e.g., type II secretion system), biofilm formation (e.g., curli, extracellular polymers) (12), secondary metabolite production (e.g., NRPS), siderophore (e.g., desferrioxamine) (13, 14), and bacteriocin biosynthesis were detected in all genomes indicating the successful adaptation to persistence and competition on marine surfaces. These genome sequences will help elucidate the mechanisms involved in *H. echinata* settlement and metamorphosis (1), and help identify novel biotechnologically important molecules.

### Nucleotide sequence accession numbers.

These whole-genome shotgun projects for strains P1-7a, P1-9, P1-13-1a, P1-16-1b, P1-25, and P1-26 have been deposited in DDBJ/EMBL/GenBank under the accession numbers LKDU00000000, LKBD00000000, LKDV00000000, LKGQ00000000, LKDW00000000, and LKDX00000000, respectively. The versions described in this paper are the first versions, LKDU01000000, LKBD01000000, LKDV01000000, LKGQ01000000, LKDW01000000, and LKDX01000000.
